# Supplementary material for: Distinct patterns of thought mediate the link between brain functional connectomes and well-being
Source: Netw Neurosci. 2020 Jul 1;4(3):637–57. doi: 10.1162/netn_a_00137 (PMC7462429; doi:10.1162/netn_a_00137)
Supplement: Supplementary file 1 [file netn-04-637-s001.pdf]

Vatansever, D., Karapanagiotidis, T., Margulies, D. S., Jefferies, E., & Smallwood, J. (2020). Supporting information for "Distinct patterns of thought mediate the link between brain functional connectomes and well-being." Network Neuroscience, *4*(3), 637–657. https://doi.org/10.1162/netn_a_00137

Supplementary Information: Distinct patterns of thought mediate the link between brain functional connectomes and well-being

Authors: Deniz Vatansever[1,2*], Theodoros Karapanagiotidis[2], Daniel S. Margulies[3], Elizabeth Jefferies[2], Jonathan Smallwood[2]

Affiliations: [1]Institute of Science and Technology for Brain-inspired Intelligence, Fudan University, Shanghai, PR China.

[2]Department of Psychology, University of York, York, UK.

[3]Brain and Spine Institute, National Centre for Scientific Research, Paris, France.

***Corresponding author:**
Deniz Vatansever, PhD

Institute of Science and Technology for Brain-Inspired Intelligence

Fudan University,

220 Handan Road, East Guanghua Building

Shanghai, PR China, 200433

Tel: +86 176 2168 7533

E-mail: deniz@fudan.edu.cn

Table of Contents

Supplementary Methods 1
Supplementary Results and Figures 2
  Decomposition of Patterns of Thoughts 2
  MRI Data Quality Assessment 8
  Network-Based Statistic 12

Supplementary Methods

Supplementary Table S1. Set of thought sampling questions administered immediately following the resting state fMRI scanning session. Participants characterized their thoughts based on a 4-point Likert scale.

Number	*Questions*	*Naming*
1	My thoughts involved future events.	Future
2	My thoughts involved past events.	Past
3	My thoughts involved myself.	Self
4	My thoughts involved other people.	Other
5	I thought about something positive.	Positive
6	I thought about something negative.	Negative
7	My thoughts were in the form of images.	Images
8	My thoughts were in the form of words.	Words
9	My thoughts were detailed and specific.	Specific
10	My thoughts tended to evolve in a series of steps.	Evolving
11	My thoughts were vivid as if I was there.	Vivid
12	My thoughts were spontaneous.	Spontaneous
13	My thoughts were deliberate.	Deliberate
14	This thought was similar to thoughts I often have.	Habitual
15	My thoughts were related to the here and now.	Here-Now
16	My thoughts were related to a more distant time.	Distant-Time
17	My thoughts were hard for me to stop.	Hard to Stop
18	My thoughts were on topics that I care about.	Important
19	My thoughts were about ideas rather than events or objects.	Abstract
20	My thoughts at different points in time were all on the same theme.	Thematic
21	My thoughts dragged my attention away from the external world.	Decoupling
22	My thoughts were intrusive.	Intrusive
23	I was thinking about an event that has happened or could take place.	Realistic
24	My thoughts gave me a new insight into something I have thought about before.	Insightful
25	I was thinking about solutions to problems (or goals).	Problem-based

Supplementary Results and Figures

Decomposition of Patterns of Thoughts

The hierarchical clustering of ratings on the 25 questions from the thought sampling questionnaire resulted in two major clusters (Fig. S1a). The similarity index (Bκ) (Fowlkes & Mallows, 1983) calculated between the original cluster membership and across 5,000 bootstrap samples illustrated reduced similarity with increasing number of clusters (Fig. S1b). Two-cluster solution showed the highest average similarity score (Bκ = .83, SE = 0.13, 95% BCI [0.63, 1]) indicating the stability of the identified clusters in this study.

The two clusters of ratings were then decomposed into patterns of thought using principal component analysis (PCA). The percentage of explained variance per component for the two clusters of ratings are provided in Figure S2a-b. The cut-off point of three components was selected based on the eigenvalue (>1) and the explanatory power gained by each additional component. The heatmaps for the component loadings of the six identified patterns of thought are displayed in Figure S3a-b, while Figure S3c-d illustrates the average component loadings across 5,000 bootstrap samples. There was high concordance in component loadings between the original and bootstrap samples as quantified via Pearson correlations for the top three components within each cluster of thought ratings (Fig. S4). In particular, the important/specific and deliberate/verbal thoughts showed relatively high correlation with the average Pearson r scores measuring 0.83 (SE = .18, 95% BCI [.34, 97]) and .96 (SE = .062, 95% BCI [.89,.99]), respectively.

Figure S5 displays typical responses from participants who scored highest on the identified thought patterns. The component scores for each individual on these patterns of thought were then carried forward on to the NBS analysis.

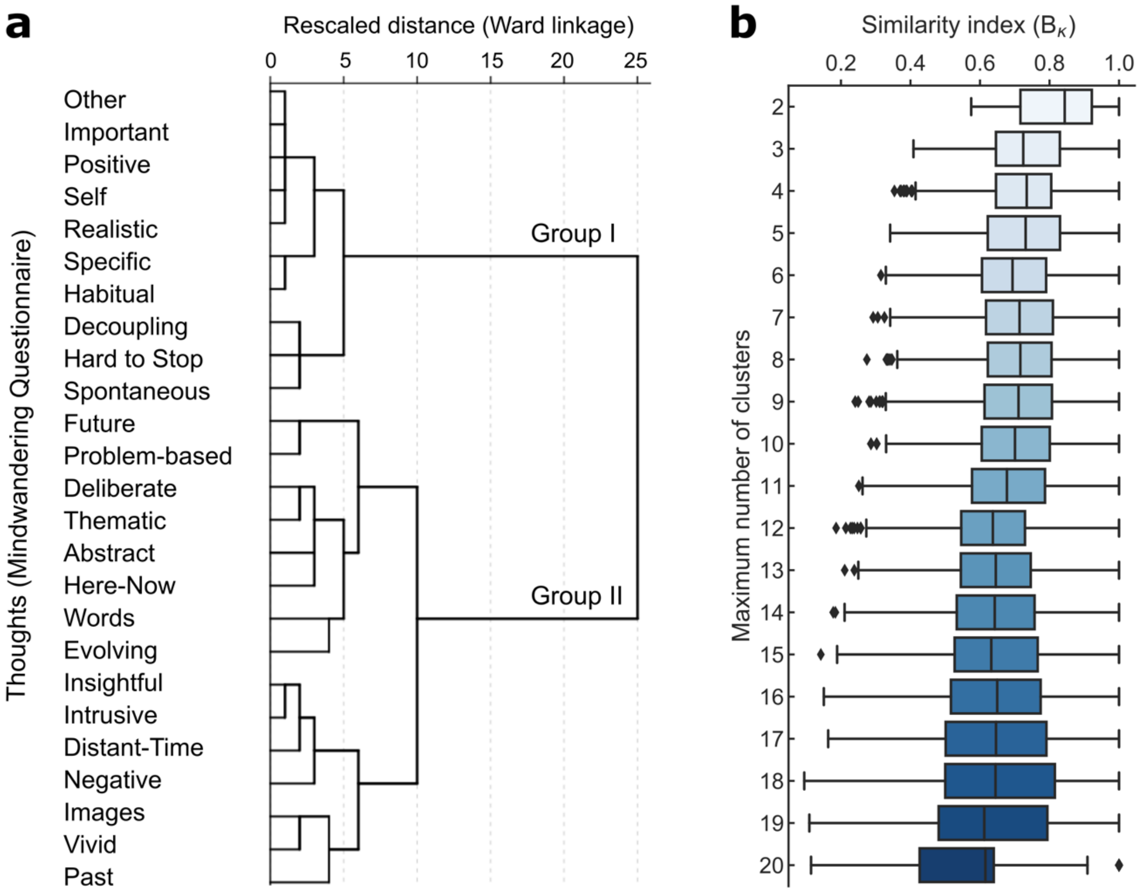

Supplementary Figure S1. Hierarchical clustering of thought sampling ratings. The participants' ratings for each question was hierarchically clustered using the Ward linkage method (squared Euclidean distance). (a) The dendrogram for the resulting clusters indicates two major clusters separated into Group I and II. (b) In order to test the reliability of the identified clusters, the similarity index (B*k*) (Fowlkes & Mallows, 1983) was calculated between the original cluster membership and those obtained across 5,000 bootstrap samples. As illustrated in traditional boxplots, results indicated reduced similarity with increasing number of clusters in which the two-cluster solution displayed the highest similarity score.

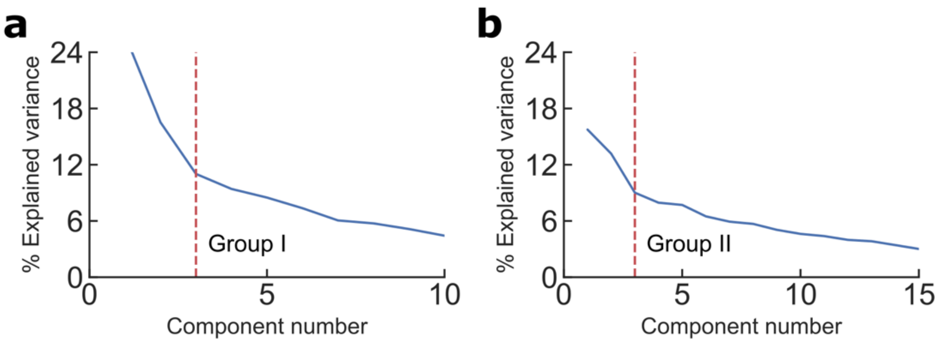

Supplementary Figure S2. Decomposition of thought patterns. The two groups of hierarchically clustered responses to the experience sampling questionnaire were decomposed into three patterns of thought each (a-b), resulting in a total of six decompositions. The eigenvalue (< 1) and the explanatory power gained by each additional decomposition (illustrated in line plots) were used to identify the cut-off point of three components.

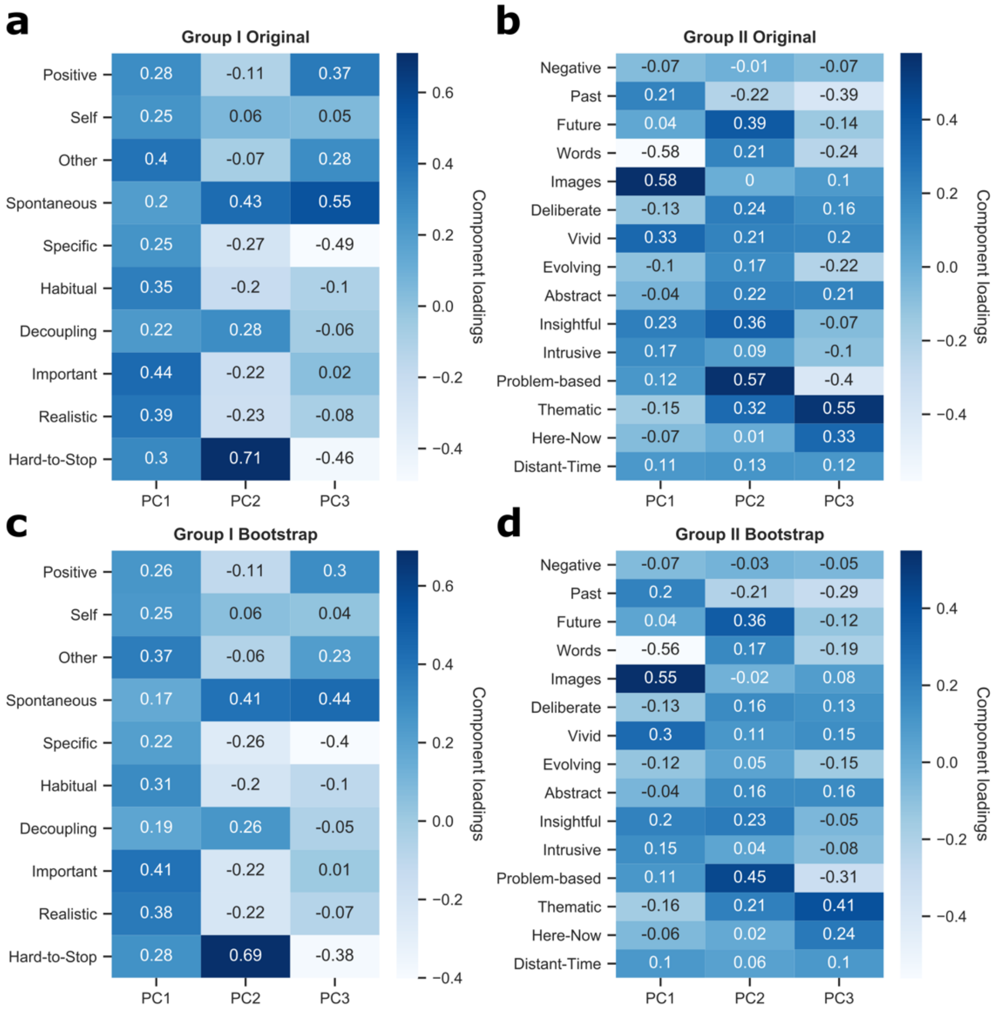

Supplementary Figure S3. Decomposition of thought patterns with bootstrap resampling. The same PCA pipeline with Varimax rotation from the main experiment was carried out across 5,000 bootstrap samples. The heatmaps illustrate loadings of the three components in each cluster of ratings for both (a-b) original and (c-d) bootstrap samples (average).

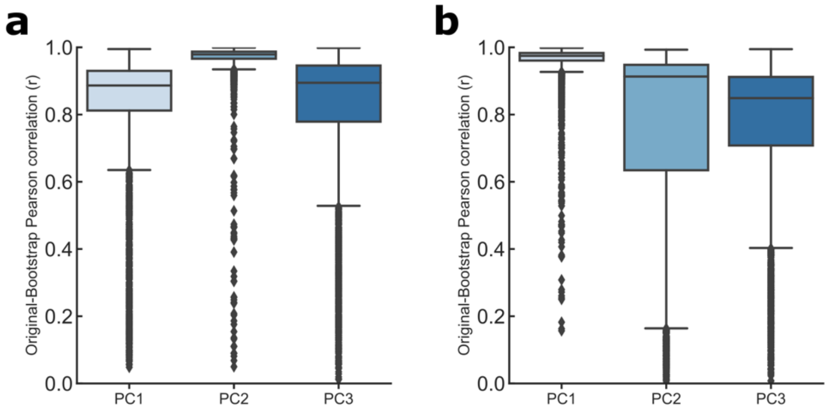

Supplementary Figure S4. Stability assessment for the decomposition of thought patterns. With the aim of investigating the stability of the identified thought patterns, Pearson correlations (r) were calculated for loadings across the three components in each group of ratings between the original and 5,000 bootstrap samples. Traditional boxplots illustrate the distribution of r values for the three components in (a) Group I and (b) Group II. Notably, the important/specific (Fig S4a, PC1) and deliberate/verbal thoughts (Fig S4b, PC2) showed relatively high correlation with the average Pearson r scores measuring 0.83 (SE = .18, 95% BCI [.34, 97]) and .96 (SE = .062, 95% BCI [.89,.99]), respectively.

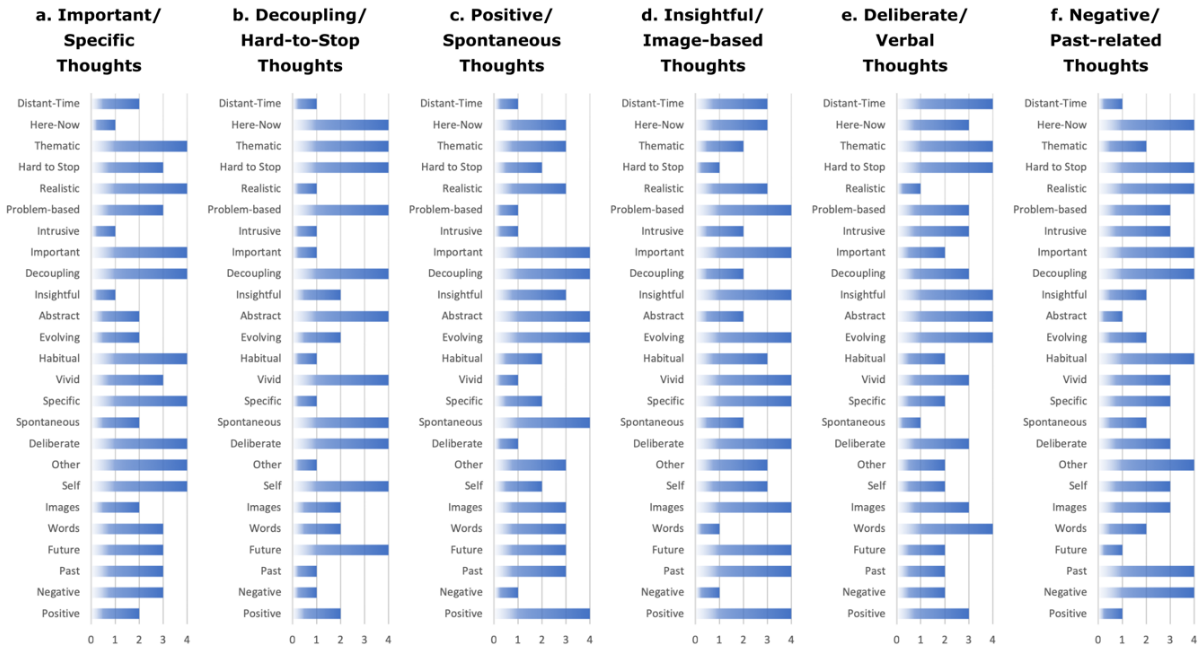

Supplementary Figure S5. Typical ratings on the thought sampling questionnaire. Bar charts illustrate raw ratings of the participants with the highest component score in the identified patterns of thought (a-f).

MRI Data Quality Assessment

The distributions of maximum and average motion parameter values, as well as the average correlation coefficients before and after the employed denoising procedures are provided in Supplementary Figure S6. Following a strict motion-correction procedure, 12 participants who had more than 15% of their data affected by motion were excluded from the analysis.

In order to further test the potential influence of in-scanner head motion on subsequent analyses, mean framewise displacement score was calculated using the Jenkinson formulation (Jenkinson, Bannister, Brady, & Smith, 2002) for all participants and MRI scanning sessions. Across the two thought patterns identified in this study, there was no significant correlation observed between the mean framewise displacement and either the component scores or the associated fractional strength measures ($p > .05$) (Fig. S7a-d). Furthermore, in addition to showing no significant difference between the two MRI scanning sessions ($t_{(39)} = -.53$, $p = .602$) (Fig. S8a), the mean framewise displacement did not illustrate any significant correlations with either the component loadings or the fractional strength measure within the two visits ($p > .05$) (Fig. S8b-e).

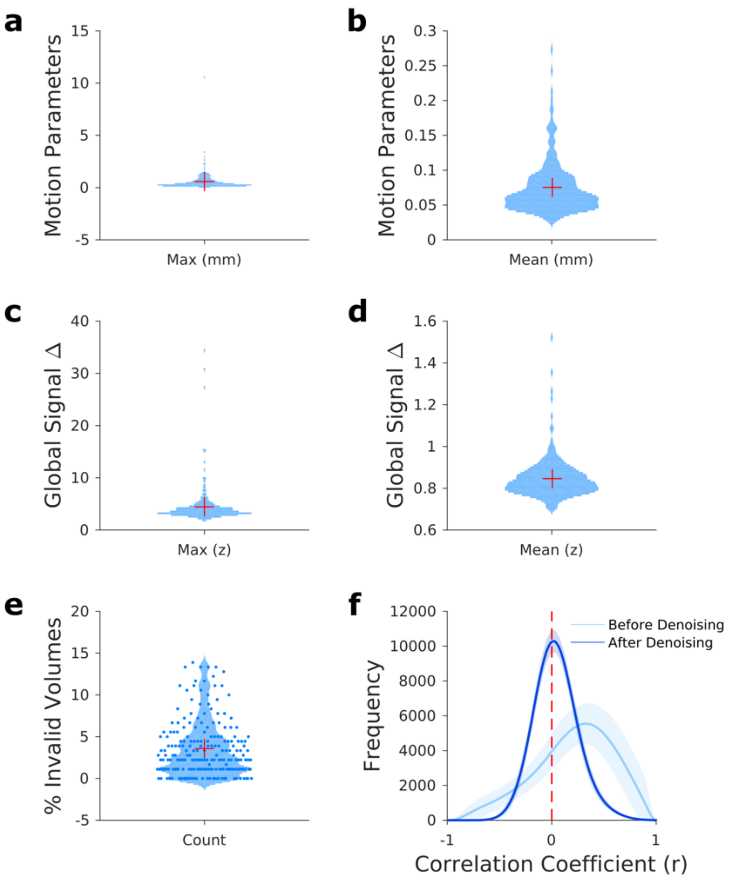

Supplementary Figure S6. MRI data quality assessment and motion correction. An extensive motion-correction procedure was employed including the removal of motion parameters and their second-order derivatives, CompCor components attributable to white matter and cerebrospinal fluid and linear detrending. In addition, the volumes associated with excessive motion were identified and scrubbed. Participants with a percentage of invalid volumes greater than 15% of their total data were excluded from the analysis. Distributions of (a-b) mean and maximum translation parameters (mm), (c-d) mean and maximum global signal change (z), and the (e) percentage of invalid scans for the final cohort of participants that were included in this analysis are provided using violin plots. The red stars indicate the 50th percentile. (f) In addition, the histogram of the average voxel-based correlation coefficients (r) across participants showed a normal distribution following the denoising steps employed in this study. The shaded areas represent standard deviation.

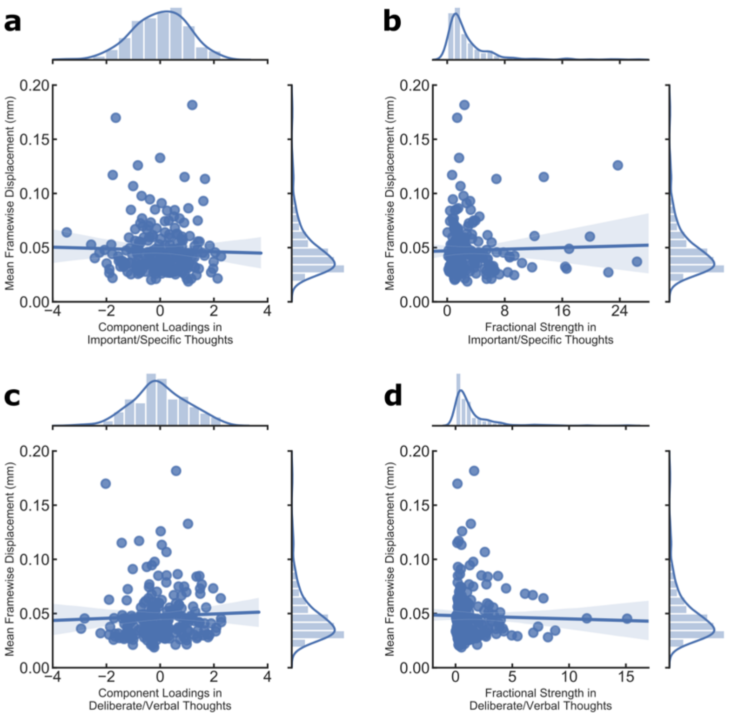

Supplementary Figure S7. Quality assurance assessment using mean framewise displacement. In order to further investigate the potential influence of in-scanner head motion on subsequent analyses, framewise displacement was calculated using the Jenkinson formulation (Jenkinson et al., 2002). Across the two identified thought patterns, no significant correlation was observed between the mean framewise displacement and either (a, c) the component scores or (b, d) the fractional strength measure employed in this study ($p > .05$).

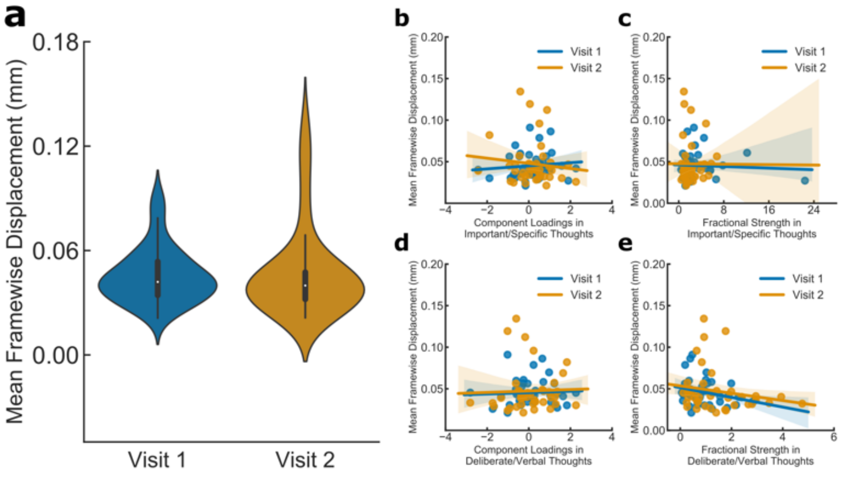

Supplementary Figure S8. Quality assurance assessment using mean framewise displacement across two MRI scanning sessions. For each participant and each visit, the mean framewise displacement was calculated using the Jenkinson et al. formulation (Jenkinson et al., 2002). (a) There was no significant difference in the mean framewise displacement between the two scanning sessions ($t_{(39)} = -.53$, $p = .60$). (b-d) Correlation analyses across the component loadings and the fractional strength measure for the two visits did not show any significant links with the mean framewise displacement ($p > .05$).

Network-Based Statistic

For the main NBS analysis, t-tests were carried out on fully connected whole-brain networks for each pattern of thought at an initial T threshold of T = 3.2 over 5,000 permutations and $p < .05$ level of significance. For the two patterns of thought which significantly related to brain connectivity components, we also provide two further analyses using T thresholds of T = 3.1 and T = 3.3. This yielded significant and comparable results to the T = 3.2 threshold reported in the main manuscript (Supplementary Fig. S9).

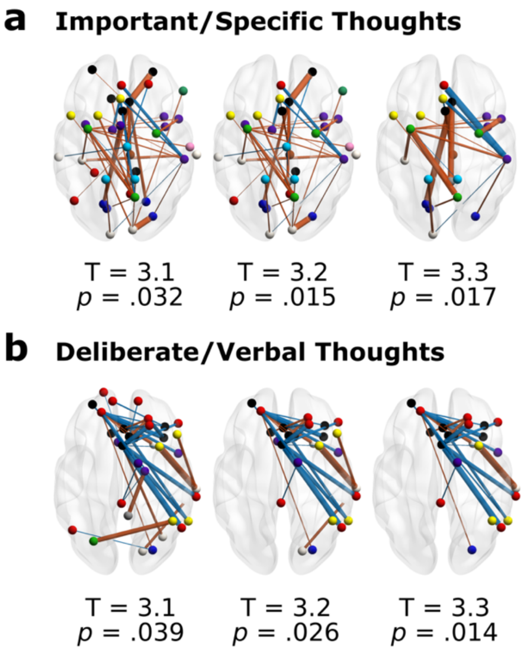

Supplementary Figure S9. Replicability of network-based statistic results at different initial T thresholds. For the two patterns of thought that significantly related to brain connectivity components, the same NBS analysis was run using two different T thresholds at T = 3.1 and T = 3.3. The resulting brain components are visualized on MNI152 smoothed brains and the corresponding *p* values of the statistical analysis for the two components (a-b) and at different thresholds are provided.

Supplementary References

Fowlkes, E. B., & Mallows, C. L. (1983). A Method for Comparing Two Hierarchical Clusterings. *Journal of the American Statistical Association, 78*(383), 553-569. doi:10.1080/01621459.1983.10478008

Jenkinson, M., Bannister, P., Brady, M., & Smith, S. (2002). Improved optimization for the robust and accurate linear registration and motion correction of brain images. *Neuroimage, 17*(2), 825-841. doi:10.1016/s1053-8119(02)91132-8
